# Exactitud diagnóstica de la técnica *Schistosoma ICT IgG-IgM* frente a otras técnicas de detección de esquistosomiasis urinaria en Nigeria

**DOI:** 10.1515/almed-2021-0005

**Published:** 2021-02-10

**Authors:** Robert Soumay Houmsou, Binga Emmanuel Wama, Hemen Agere, John Ador Uniga, Timothy Jerry Jerry, Paul Azuaga, Elizabeth Une Amuta, Santaya Larit Kela

**Affiliations:** Departamento de Ciencias Biológicas, Taraba State University, Jalingo, Nigeria; Departamento de Ciencias Biológicas, Federal University Wukari, Wukari, Nigeria; Unidad de Pediatría, Federal Medical Centre, Jalingo, Taraba, Nigeria; Departamento de Zoología, Federal University of Agriculture, Makurdi, Benue State, Nigeria; Departamento de Ciencias Biológicas, Federal University Kashere, Kashere, Gombe State, Nigeria

**Keywords:** comparación, esquistosomiasis, evaluación, Nigeria, urinaria

## Abstract

**Objetivos:**

La esquistosomiasis es una enfermedad parasitaria causada por gusanos del género esquistosomazonas que afecta a humanos en zonas rurales donde el parásito es endémico. En este estudio se evalúa el rendimiento diagnóstico del test rápido *Schistosoma ICT IgG-IgM* para la detección de la esquitosomiasis urinaria en Nigeria.

**Métodos:**

Se analizaron un total de 374 muestras de orina. Para el análisis de las muestras se emplearon tiras reactivas, filtración de orina y el test de *Schistosoma ICT IgG-IgM*. Se emplearon 2 mL de suero para el análisis serológico con el test *Schistosoma ICT IgG-IgM*. A continuación, se enviaron 3 mL de cada muestra sérica a LDBIO Diagnostics (Francia) para un segundo análisis con el test *Schistosoma ICT IgG-IgM* y confirmación mediante IgG SCHISTO *Western blot* (WB). El rendimiento de la prueba evaluada se determinó calculando la sensibilidad (Se), especifidad (Sp), valor predictivo positivo (VPP), valor predictivo negativo (VPN) y la razón de verosimilitud positiva (LR+). La exactitud de cada prueba se determinó calculando el Índice de Youden (IY) y la exactitud diagnóstica (ED). El nivel de significación estadística se determinó en un valor p≤0,05. El test *Schistosoma ICT IgG-IgM* detectó la infección en el 63,9% de las muestras.

**Resultados:**

El test mostró una sensibilidad del 94,9%, una especifidad del 63,9%, un valor predictivo positivo del 72,4%, un valor predictivo negativo del 92,6% y una razón de verosimilitud de 2,62. La técnica *Schistosoma ICT IgG-IgM* mostró un buen índice kappa de Cohen (κ=0,68), un buen Índice de Youden (IY=0,58), así como una buena exactitud diagnóstica (ED=0,78).

**Conclusiones:**

*Schistosoma ICT IgG-IgM* ha demostrado ser la mejor técnica para la detección de la esquistosomiasis urinaria en Nigeria.

## Introducción

La esquistosomiasis es una enfermedad tropical olvidada causada por la especie *Schistosoma*, que son gusanos trematodos. La enfermedad es una patología crónica aguda que afecta a los habitantes de zonas rurales. Se estima que actualmente hay unos 240 millones de personas en todo el mundo infectadas, y que el 90% de ellas viven en el África subsahariana [1]. En esta región, la esquistosomiasis representa un serio problema de salud pública para los habitantes de las zonas rurales. Por lo general, estas personas se exponen a la infección por las prácticas agrícolas empleadas en el cultivo de arroz, así como por sus actividades recreativas y domésticas y por pescar y bañarse en lagos o ríos infestados [2], [ 3]. En Nigeria, la esquistosomiasis urinaria afecta a los habitantes de las zonas rurales. Según la zona geográfica, su incidencia varía entre el 62,0% en el estado de Kwara y el 45,6% en el estado de Kano [4], [ 5]. También se ha detectado *Schistosoma mansoni* en habitantes de zonas rurales, aunque con una baja tasa de infección entre las diferentes comunidades [6], [ 7].

La detección rutinaria de la esquistosomiasis se suele realizar en centros sanitarios de zonas rurales y urbanas empleando tiras reactivas [8], [ 9]. En el pasado, la esquistosomiasis urinaria se solía diagnosticar mediante la filtración de orina y la visualización de huevos. Sin embargo, el rendimiento de estas pruebas no resultaba satisfactorio, ya que su eficacia es limitada a la hora de detectar únicamente esta enfermedad en pacientes infectados; además son técnicas complejas y laboriosas [10].

Recientemente se ha desarrollado una técnica rápida cualitativa, *Schistosoma ICT IgG-IgM*. En este estudio se evalúa el rendimiento diagnóstico de la prueba *Schistosoma ICT IgG-IgM* en Nigeria seis meses después de la aprobación de su comercialización. En el presente estudio se compara el rendimiento diagnóstico de *Schistosoma ICT IgG-IgM* con el de las tiras reactivas y la filtración de orina, que son las técnicas empleadas rutinariamente en los centros sanitarios.

## Materiales y métodos

### Área del estudio

Este estudio se realizó en el Área de Gobierno Local (LGA) de Takum, localizada en latitud 07° 15′N y longitud 09° 59′E del Estado de Taraba, en el noreste de Nigeria. Limita al sur con la República de Camerún, al oeste con el LGA de Ussa, al con el LGA de Donga, y al este con el LGA de Katsina-Ala, en el estado de Benue. El Área de Gobierno Local se divide en 10 distritos, de los cuales seleccionamos 7. La selección se basó en la presencia de Centros de Atención Primaria a los que se deriva a los pacientes para la administración de tratamiento. Todas las áreas cuentan con la presencia de ríos caudalosos de los que depende la vida diaria de sus habitantes. Los distritos seleccionados fueron Barki-Lissa, Birama, Chanchanji, Kashimbila, Manya, Malumshe y Sufa (Material Suplementario 1).

El clima del área es tropical, con la vegetación típica de la sabana guineana intercalada de bosque ripario. Existen dos estaciones: la estación de lluvias, que comienza a mediados de marzo y culmina a mediados de octubre, y la estación seca, de mediados de octubre a mediados de marzo. Las precipitaciones oscilan entre 1.200 y 2.000 mm. La temperatura media se encuentra entre los 24 y los 32 °C, con un máximo de 37 °C en marzo y abril.

El área está habitada por funcionarios, intermediarios del sector agrícola y agricultores. Esta área es una zona de intensa actividad agrícola y de pesca.

### Diseño del estudio

Se realizó un estudio prospectivo sobre el uso de la técnica *Schistosoma ICT IgG-IgM* sobre el terreno. Se utilizaron la filtración de orina, la microhematuria y la proteinuria como pruebas índices. Las muestras serológicas se enviaron a LDBIO Diagnostics (Lyon, Francia) para un segundo análisis con la prueba *Schistosoma ICT IgG-IgM* y confirmación mediante IgG SCHISTO *Western blot* (WB).

### Aprobación ética

Previamente al inicio del estudio, se obtuvo la autorización por parte de la Delegación de Salud del LGA de Takum (TLG/PHC/403) y se presentó el estudio ante el Comité Ético del Centro Médico Federal en Jalingo, en el estado de Taraba.

### Elegibilidad de los participantes

En los tres días anteriores al inicio de la toma de muestras, nos pusimos en contacto con la autoridad local de cada distrito para explicarles la importancia del estudio A la hora de tomar las muestras de orina y sangre, nos comunicamos con los pacientes en lengua “*jukun*”, la lengua local de la zona. El día antes de iniciar la toma de muestras de orina y heces, se pidió a los habitantes de cada distrito que se reunieran en el principal espacio de reuniones de la localidad. Se solicitó a cada participante autorización para que su centro de atención primaria recogiera las muestras. Nos comunicamos con los adultos, los niños y sus padres o tutores en su lengua tribal el ‘*jukun*’. Los participantes siguieron las instrucciones que se les había dado antes de la obtención de las muestras.

### Tamaño de la muestra: Criterios de inclusión y exclusión

El tamaño de la muestra se calculó con la siguiente fórmula:

$$N=\frac{Z^{2}\cdot P\left(1-P\right)\cdot D}{E^{2}}$$

donde *P*=50% es la prevalencia estimada de esquistosomiasis urinaria. *D*=1 refleja el efecto de diseño de la aleatorización simple empleada para el reclutamiento de pacientes. E^2^=0,0025, precisión (margen de error, que es el 10% de la prevalencia estimada). *Z*=1,96, para dos colas.

En total, se incluyeron 420 pacientes en el estudio. Se tomaron muestras de 60 individuos de cada uno de los siete distritos. Se excluyó a los pacientes que habían estado enfermos o con patologías del sistema urinario en las dos últimas semanas. También se excluyó a las mujeres que estuvieran menstruando y a los sujetos que se negaron a que se les extrajeran 5 mL de sangre para la obtención de plasma. Los sujetos que se negaron a entregar 10 mL de orina para la filtración de la misma también fueron excluidos. Algunos individuos se negaron a participar debido a creencias culturales sobre el uso de la sangre y la orina. Se incluyó en el estudio a todos los participantes que cumplieron los criterios para la toma de una muestra de sangre y orina. A los participantes a los que se les diagnosticó esquistosomiasis urinaria se les trató inmediatamente con Praziquantel.

### Análisis clínicos

Se les explicó a los participantes y progenitores de los niños los métodos empleados en las diferentes pruebas analíticas. Para el análisis de orina, se utilizó la filtración de orina y el uso de tiras reactivas, mientras que para el análisis de muestras fecales se utilizó la técnica de Kato-Katz (Sterlitech Corporation, Kent, EE. UU). El examen serológico se realizó con la técnica *Schistosoma ICT IgG-IgM*. Los duplicados de suero anonimizados y codificados obtenidos en el estudio de campo se enviaron a LDBIO Diagnostics (Lyon, Francia) para un segundo análisis con *Schistosoma ICT IgG-IgM.* El diagnóstico se confirmó con la prueba SCHISTO II WB IgG.

### Determinación sobre el terreno de microhematuria and proteinuria

La microhematuria (Ery/μL) y la proteinuria (mg/dL) se determinaron mediante el uso de tiras reactivas (Medi-Test Combi 9, MACHERY-NAGEL, Alemania). Los resultados para microhematuria (Ery/μL) y la proteinuria (mg/dL) se interpretaron de la siguiente manera: 0=negativo, Ca.10=+, Ca.50=++ y Ca.100=+++. La proteinuria se midió como: 0=negativo, Ca.30=+, Ca.100=++ y Ca.500=+++.

### Técnicas de filtración y de Kato-Katz

La filtración es una técnica de campo que se utiliza para el diagnóstico de esquistosomiasis urinaria. Para la filtración de orina se emplearon una jeringa de 10 mL, portafiltros de polipropileno Swinney (13 mm de diámetro) y filtros de membrana de policarbonato (porosidad de 12,0 μm) (Sterlitech Corporation, Kent, EE. UU). Los resultados de la filtración de orina se interpretaron de la siguiente manera: 0 huevos/10 mL de orina (ausencia de infección), 1–49 huevos/10 mL de orina (infección leve) y >50 huevos/10 mL de orina (infección grave) aplicando las guías de la OMS [11].

Los análisis fecales se realizaron aplicando las técnicas de Kato-Katz y de concentración fecal. La infección se clasifica como <100 huevos por gramo (carga parasitaria baja), 100–399 huevos por gramo (carga parasitaria media) *y*>400 huevos por gramo (carga parasitaria alta) [11].

### Schistosoma ICT IgG-IgM

Recientemente se ha desarrollado una técnica rápida de análisis cualitativo, *Schistosoma ICT IgG-IgM* Esta técnica está basada en tecnología de inmunocromatografía, mediante la cual se detectan simultáneamente anticuerpos IgG e IgM. Se extrajo una muestra de 10 mL de sangre de cada sujeto. A continuación, se prepararon 5 mL de suero de cada muestra de sangre. Para cada test de *Schistosoma ICT IgG-IgM* se emplearon 30 mL de suero preparado para el análisis de esquistosomiasis. El resultado positivo, negativo o inválido se determinó mediante un test en cassette cuya duración es de 20–30 minutos. A continuación, se describe el procedimiento: en primer lugar, se dispensaron 30 μL de suero y un eluyente en el pocillo de la muestra de cada prueba. El resultado de la prueba era positivo si aparecía una “T” roja, mientras que si aparecía una “C” azul, el resultado era negativo. La prueba no era válida si no aparecía ni una “T” ni una “C”. En las instrucciones se puede encontrar una descripción del procedimiento de la prueba *Schistosoma ICT IgG-IgM*.

Los otros 3 mL se conservaron en 5 mL de tubos eppendorf. Se añadieron al suero 30 μL de azida sódica (NaN_3_) a una concentración de 0,02% (m/v). Una vez en el laboratorio, el suero se conservó durante 3 días en un congelador a −4 °C. Posteriormente, se envió a LDBIO Diagnostics, (Lión, Francia) para el segundo análisis con la técnica *Schistosoma ICT IgG-IgM.* Para confirmar el resultado, se empleó el método SCHISTO II WB IgG [12]. Los resultados obtenidos con la técnica *Schistosoma ICT IgG-IgM* sobre el terreno se tuvieron en cuenta en este estudio, ya que se observaron diferencias mínimas entre resultados en el segundo análisis.

### SCHISTO II *Western blot* (WB) IgG

SCHISTO II WB IgG es una técnica cualitativa de WB. Esta técnica está basada en la serología de IgG mediante un test de inmunotransferencia. Se utilizó para confirmar el resultado positivo o inequívoco obtenido con *Schistosoma ICT IgG-IgM.* En el procedimiento se utilizan geles de acrilamida (13%) y tampón de dodecilsulfato de sodio discontinuo, que provoca la electroforesis de la solución de antígenos de *Schistosoma mansoni* y *Schistosoma haematobium* Para calcular el peso molecular se emplea una mezcla de proteínas biotiniladas y previamente sometidas a tinción (miosina, *β*-galactosidasa, fosforilasa *b*, albúmina de suero bovino, ovoalbúmina, anhidrasa carbónica, inhibidor de tripsina, lisozima y aprotinina). Los geles se mueven más rápidamente que el inhibidor de tripsina 30-kDa (previa tinción con azul de metileno), que se desplaza a la parte inferior de los geles [13]. A continuación, se transfieren las proteínas a membranas nitrocelulosa con leves modificaciones, que han sido previamente recubiertas con Tris-NaCl (pH 7.4) y que contienen un 5% de leche desnatada [14]. Los *blots* se lavan dos veces con Tris-NaCl, se secan y separan en amplias tiras de 4 mm. Las tiras de antígenos se incuban con un tampón de suero diluido (1:50 in Tris-NaCl) durante 90 minutos. A continuación, se incuban las tiras con un conjugado de fosfatasa G-alcalina durante 60 minutos, tras una fase de lavado con un tampón de lavado de Tris-NaCl. A continuación, se vuelven a lavar las tiras. El suero reconoce las fracciones de proteínas mostradas por el sustrato cromogénico, que contiene nitroazul de tetrazolio y 5-bromo-4-cloro-3-indolilfosfato. Las tiras se lavan con agua destilada para detener la reacción. Se secan las tiras y se fijan en papel para su lectura y almacenamiento. Se verificaron tanto los resultados positivos como los negativos de cada prueba.

### Análisis de datos

Los datos se recopilaron y clasificaron con Microsoft Excel. Con XLSTAT 2017 se determinó la sensibilidad (Se), especifidad (Sp), valor predictivo positivo (VPP), valor predictivo negativo (VPN) y la razón de verosimilitud positiva (LR+) de las pruebas. El Índice de Youden (IY) de cada prueba se calculó como sensibilidad + especifidad-1. La exactitud diagnóstica (ED) se calculó como ED=Positivo verdadero + negativo verdadero/positivo verdadero + negativo verdadero + falso positivo + falso negativo. La índice kappa de Cohen y la concordancia entre las pruebas índice se calcularon con el programa IBM SPSS 23.0. Los valores del índice kappa de Cohen (κ) se interpretaron de la siguiente forma: <0,20 (poca concordancia); 0,20–0,40 (regular concordancia); 0,41–0,60 (concordancia moderada); 0,61–0,80 (buena concordancia) y 0,81–1,00 (muy buena concordancia). Se emplearon un intervalo de confianza (IC) del 95% y un valor p≤0,05 para establecer la significación estadística de las pruebas.

## Resultados

### Diagrama de flujo de participantes

La edad de los participantes oscilaba entre los 3 y los 51 años (12,8 ± 10,7). Los hombres representaron un 46,2% del total, mientras que las mujeres representaron un 53,7% del total de la muestra.

**Figura 1:**
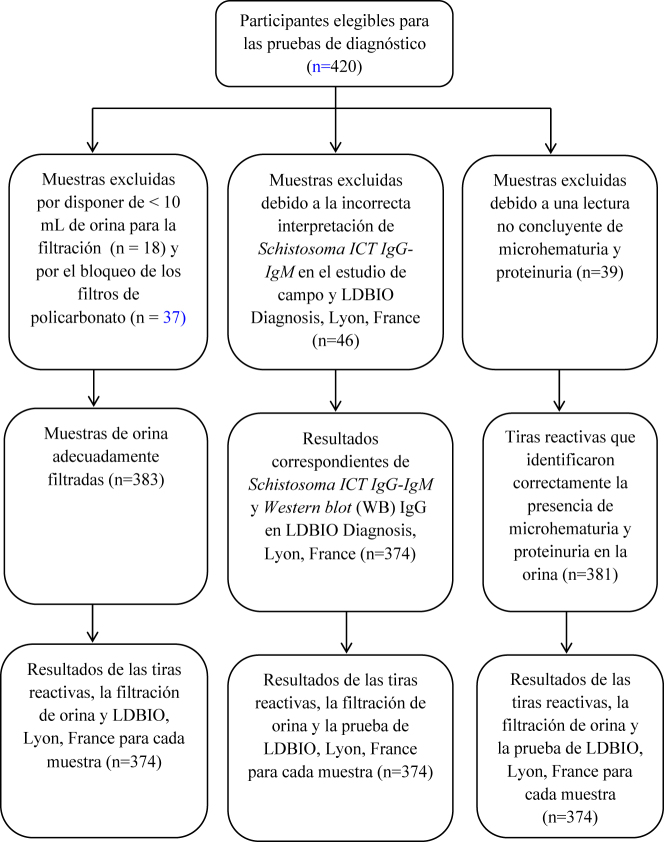
Flujo de pacientes (inclusión y exclusión).

En la Figura 1 se muestra el flujo de participantes en el estudio. Se les explicaron los criterios para la toma de muestras a los participantes. A continuación, se seleccionó aleatoriamente a 60 individuos de cada distrito. En total, 420 participantes fueron elegibles para el estudio. Un total de 46 pruebas de *Schistosoma ICT IgG-IgM* no fueron aceptadas debido a discordancias entre los técnicos de laboratorio. Se excluyeron 37 muestras de orina porque se disponía de <10 mL de orina para la filtración (n=18) o por la no filtración de los filtros de policarbonato (n=19). Algunas tiras reactivas dieron resultados no concluyentes de proteinuria (n=21) y microhaematuria (n=18).

Finalmente, 374 muestras dieron resultados concordantes entre las tiras reactivas, la filtración, *Schistosoma ICT IgG-IgM* y SCHISTO II *Western blot* (WB) IgG.

### Análisis fecal

En ninguna de las muestras fecales examinadas se detectó infección por *S. mansoni*. En algunas muestras se hallaron parásitos intestinales, por lo que fueron excluidos del estudio. Estos fueron *Trichuris trichiura* (12,2%), *Ascaris lumbricoides* (8,2%), *Enterobius vermicularis* (6,1%), *Taenia* sp (4,8%) y *Strongyloides stercoralis* (3,2%)*.*

### Concordancia de resultados positivos entre las tests índice (microhematuria, proteinuria, filtración, y *Schistosoma ICT IgG-IgM*) y la técnica estándar oro de WB IgG

En la Tabla 1 se muestra la concordancia entre las pruebas índice y el WB IgG estándar. El test que dio menos positivos fue la microhaematuria (6,1%) mientras que *Schistosoma ICT IgG-IgM* fue la que arrojó mayor número de resultados positivos (44,9%). Entre las pruebas *Schistosoma ICT IgG-IgM* y la prueba estándar oro *Western blot* (WB) *IgG immunoblot* son las que mostraron una mayor concordancia según la índice Kappa de Cohen (κ=0,68). Los resultados de la filtración no mostraron un buen índice de Cohen (κ=0,04) y no concordaban con los resultados de la prueba WB IgG.

**Tabla 1:** Concordancia de resultados positivos entre las pruebas índice (microhematuria, proteinuria, filtración, y Schistosoma ICT IgG-IgM) y la técnica (estándar oro) de *Western blot* (WB) IgG.

	WB IgG		Total	Kappa	
	Negativa	Positiva			
				de Cohen (κ)	[IC95%]
Microhematuria				0*,*05	0*,*02–0*,*08
Negativa, n (%)	181 (48*,*4)	154 (41*,*2)	335 (89*,*6)		
Positiva, n (%)	16 (4*,*3)	23 (6*,*1)	39 (10*,*4)		
Total, n (%)	197 (52*,*7)	177 (47*,*3)	374		
Proteinuria				0*,*13	0*,*07–0*,*32
Negativa, n (%)	117 (31*,*3)	81 (21*,*7)	198 (52*,*9)		
Positiva, n (%)	80 (21*,*4)	96 (25*,*7)	176 (47*,*1)		
Total, n (%)	197 (52*,*7)	177 (47*,*3)	374		
Filtración				0*,*04	0*,*01–0*,*07
Negativa, n (%)	136 (36*,*4)	114 (30*,*5)	250 (66*,*8)		
Positiva, n (%)	61 (16*,*3)	63 (16*,*8)	124 (33*,*2)		
Total, n (%)	197 (52*,*7)	177 (47*,*3)	374		
Esquistosoma				0*,*68	0*,*41–0*,*83
*ICT IgG-IgM*					
Negativa, n (%)	126 (33*,*7)	9 (2*,*4)	135 (36*,*1)		
Positiva, n (%)	71 (19*,*0)	168 (44*,*9)	239 (63*,*9)		
Total, n (%)	197 (52*,*7)	177 (47*,*3)	374		

Información secundaria: κ≤0, discordancia; κ=0–0,20, baja concordancia; κ=0,21–0,40, regular concordancia; κ=0,41–0,60, concordancia moderada; κ=0.61–0.80, buena concordancia; κ=0.81–1.00, muy buena concordancia.

### Rendimiento diagnóstico de las pruebas empleadas para la detección de esquistosomiasis urinaria en zonas rurales del Área de Gobierno Local de Takum, en el estado de Taraba (Nigeria)

La prueba *Schistosoma ICT IgG-IgM* fue la que mostró tener mayor sensibilidad (94,9%) y una especifidad bastante buena (63,9%). Además, esta prueba fue la que mostró mayor valor predictivo positivo (72,3%), mayor valor predictivo negativo (92,6%) y mayor razón de verosimilitud positiva (2,62). Así mismo, también obtuvo el Índice de Youden más alto (0,58) y la mayor exactitud diagnóstica (0,78) (Tabla 2).

**Tabla 2:** Rendimiento diagnóstico de las pruebas empleadas para la detección de esquistosomiasis urinaria en zonas rurales del Área de Gobierno Local de Takum, Estado de Taraba (Nigeria).

	Se, %	Sp, %	VPP, %	VPN, %	LR+	IY	ED
Pruebas diagnósticas	(IC 95%)	(IC 95%)	(IC 95%)	(IC 95%)	(IC 95%)		
Micro-hematuria	12,9 (0,08–18,8)	91,8 (87,1–94,9)	61,5 (46,2–76,8)	51,3 (46,0–56,7)	1,57 (0,87–2,92)	0,04	0,54
Proteinuria	54,2 (46,8–61,4)	59,3 (52,4–66,0)	57,1 (49,8–64,4)	56,4 (49,5–63,3)	1,33 (1,07–1,65)	0,13	0,56
Filtración	35,5 (28,9–42,9)	69,0 (62,2–75,0)	53,4 (44,7–62,2)	51,7 (45,5–57,9)	1,14 (0,86–1,53)	0,04	0,53
Esquistosoma *ICT IgG-IgM*	94,9 (90,4–97,4)	63,9 (56,8–70,1)	72,4 (66,8–78,1)	92,6 (88,1–97,0)	2,62 (2,16–3,16)	0,58	0,78

Se, sensibilidad; Sp, especifidad; VPP, valor predictivo positivo; VPN, valor predictivo negativo; LR+, razón de verosimilitud positiva (LR+); IY, Índice de Youden; ED, exactitud diagnóstica; IC, ntervalos de confianza.

## Discusión

Determinar la endemicidad de la esquistosomiasis urinaria en zonas rurales es muy importante. La prueba *Schistosoma ICT IgG-IgM* fue la que mostró mayor razón de verosimilitud positiva, mostrando mejor concordancia con la prueba estándar oro de WB IgG que las otras pruebas empleadas en el estudio. La baja concordancia entre algunas pruebas evidenció la cautelosa actitud de los técnicos de laboratorio que determinaban erróneamente la presencia de esquistosomiasis en individuos infectados de las zonas rurales. En el presente estudio, la técnica *Schistosoma ICT IgG-IgM* se postula como una prueba de detección de la esquistosomiasis más útil y precisa que las demás. El uso de la prueba *Schistosoma ICT IgG-IgM* evitará la falta de confianza de la población rural hacia los análisis clínicos para la detección de patologías en los centros de atención primaria. Estos resultados concuerdan con los obtenidos en estudios previos en los que se utilizó la prueba *Schistosoma ICT IgG-IgM* para detectar la enfermedad en personas que residían ilegalmente en islas de Italia en su viaje hacia otros países europeos [15], [ 16]. La proteinuria fue la técnica que mostró una segunda mejor concordancia, lo que podría deberse al efecto de los huevos de *S. haematobium* sobre los glomérulos, lo que provoca la excreción de albúmina en orina, lo que indica la presencia de esquistosomiasis urinaria.

Aunque el uso de la microhematuria y la proteinuria en las tiras reactivas está muy extendido, existen estudios que muestras resultados poco satisfactorios [17], [ 18]. El rendimiento diagnóstico de la microhematuria y la proteinuria varía significativamente, dependiendo del clima de la región africana en cuestión. En algunas partes de África, un clima más seco provoca una mayor temperatura, causando una mayor deshidratación en las personas [19]. La mayor temperatura (36–38 °C) observada en Chanchanji, Manya, Malumshe y Sufa durante la sesión seca habrían contribuido a una mayor concentración de huevos en la vejiga, lo que provoca hematuria y proteinuria. Las temperaturas más bajas (24–30 °C) observadas en las zonas con bosque ripario (Birama, Barki-Lissa, Kashimbila) provoca una menor inflamación de la vejiga urinaria. Los habitantes de estas áreas orinan con mucha mayor frecuencia, mostrando así una menor concentración de huevos en la vejiga.

La proteinuria tiene una sensibilidad mediana, mientras que la microhematuria tiene una sensibilidad baja. Las sensibilidades media y baja de estas pruebas muestran su incapacidad para detectar esquistosomiasis urinaria en áreas endémicas. En un estudio realizado en la región central de Ghana se observaron sensibilidades similares [20]. La mayor especifidad observada en la microhematuria y la filtración podrían deberse a la presencia de esquistosomas inmaduras, lo que provoca menos síntomas urinarios a los individuos infectados. La técnica *Schistosoma ICT IgG-IgM* tiene una buena sensibilidad, bastante buena especifidad y bastante buena razón de verosimilitud positiva. Esto ocurrió debido a la buena lectura de los cassettes empleados por los investigadores que ayudaron a los técnicos de laboratorio. Los estudios sobre la prueba *Schistosoma ICT IgG-IgM* en Nigeria muestran que esta es una prueba de detección rápida y eficaz en las zonas rurales endémicas. La inmunología de los individuos examinados podría haber contribuido también a los falsos negativos y falsos positivos observados. Es necesario desarrollar nuevas y mejores pruebas inmunológicas para alcanzar una especifidad muy buena.

Las técnicas inmunológicas son más eficaces que las técnicas parasitológicas empleadas en el laboratorio. Estas permiten la detección temprana de la esquistosomiasis tan solo 14 días después de la infección [21]. La efectividad a la hora de determinar los anticuerpos de los antígenos de esquistosomas en individuos infectados superó a la de la visualización de huevos mediante microscopía. Los anticuerpos juegan un papel esencial en la actividad inmune tras la infección por esquistosómulas. La inmunoglobulina M (IgM) y la Inmunoglobulina G (IgG) se generan en cuanto se produce la infección para luchar contra las esquistosómulas e inmunizar al organismo. La esquistosomiasis se determina en los individuos infectados mediante la detección de IgG e IgM en la prueba *Schistosoma ICT IgG-IgM*. Esto asegura la prescripción de Praziquantel para tratar la esquistosomiasis en los individuos infectados. Estos individuos adquirirán suficientes anticuerpos frente a una futura infección durante al menos dos años [22]. El tratamiento de la esquistosomiasis suele desencadenar respuestas inmunes humorales y celulares, que principalmente son anticuerpos frente a los esquistosomas [23]. Los diferentes anticuerpos desempeñan varias funciones a la hora de proteger a los individuos infectados frente a los esquistosomas. El papel de la IgG2 e IgG3 como supresoras de las esquistosómulas en presencia de eosinófilos activados es bien conocido. También se ha sugerido el papel modulador de IgG4 de las respuestas anafilácticas asociadas a IgE [24]. Los anticuerpos como IgE, IgM e IgG1 han demostrado ser resistentes a la esquistosomiasis [25], mientras que la IgA reduce la fecundidad de los esquistosomas [22], [ 25]. Chisango et al. [22] observaron estos beneficios de la inmunidad protectora tras la administración de Praziquantel a escolares en un control quimioterápico de la esquistosomiasis en Zimbabue.

La prueba *Schistosoma ICT IgG-IgM* tiene unos valores predictivos positivos y negativos razonablemente buenos. Así mismo, también tiene un buen índice kappa de Cohen, bastante buen Índice de Youden, y es la técnica con mejor exactitud diagnóstica. La técnica *Schistosoma ICT IgG-IgM* es la más sencilla y eficaz a la hora de detectar la esquistosomiasis en áreas endémicas del África subsahariana.

En términos económicos, la técnica *Schistosoma ICT IgG-IgM* resulta costosa para los países del África subsahariana. La empresa propietaria de esta técnica debería reducir su precio para estos países, lo que les permitirá adquirir estos kits y lograr una rápida detección. También ayudará a establecer un tratamiento a los individuos infectados de las áreas endémicas. La mayoría de los habitantes de los países del África subsahariana no tienen capacidad económica para adquirir estos kits de detección si estos tienen un coste elevado. No se puede negar la pobreza de esta población. Las diferentes empresas del mundo deberían fabricar y vender estos kits a un precio asequible.

La limitación de la prueba *Schistosoma ICT IgG-IgM* es que precisa la preparación de plasma o suero. Los habitantes se negaban a la realización de la prueba al observar a los técnicos de laboratorio extraer sangre para obtener plasma o suero. Los habitantes de las zonas rurales se oponen a la extracción de sangre por motivos culturales. En segundo lugar, el kit de análisis por inmunocromatografía tiene un precio elevado.

*Schistosoma ICT IgG-IgM* es una prueba diagnóstica adecuada y eficaz a la hora de detectar la esquistosomiasis en los países del África subsahariana. Esta técnica tiene una buena sensibilidad, bastante buena especifidad y bastante buena razón de verosimilitud positiva. El kit tiene un Índice de Youden bastante bueno y una mejor exactitud diagnóstica. Se recomienda al fabricante, LDBIO Diagnostics (Lyon, Francia), así como a otras empresas, que desarrollen pruebas similares en las que se emplee sangre en lugar de suero.

## Supplementary Material

Supplementary Material

## Material Suplementario

La versión en línea de este artículo ofrece material suplementario (https://doi.org/10.1515/almed-2021-0005).
