# Supplementary figures and images for: Exactitud diagnóstica de la técnica Schistosoma ICT IgG-IgM frente a otras técnicas de detección de esquistosomiasis urinaria en Nigeria
Source: Adv Lab Med. 2021 Feb 10;2(1):79–86. [Article in Spanish] doi: 10.1515/almed-2021-0005 (PMC10197490; doi:10.1515/almed-2021-0005)

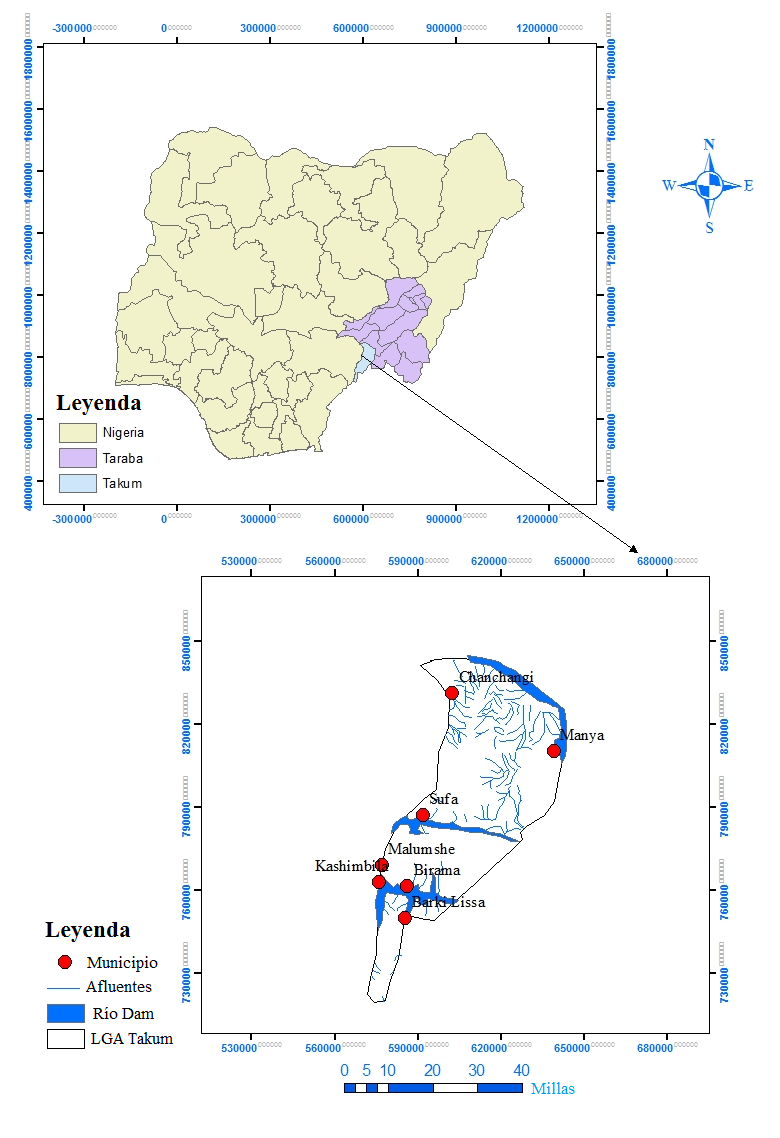


**Figura suplementaria 1**: Mapa del área del estudio

Supplement: Supplementary file 1 — Supplementary Material [file j_almed-2021-0005_suppl.docx]
